# Efficiency Enhancement of Perovskite Solar Cells by Pumping Away the Solvent of Precursor Film Before Annealing

**DOI:** 10.1186/s11671-016-1467-9

**Published:** 2016-05-12

**Authors:** Qing-Yang Xu, Da-Xing Yuan, Hao-Ran Mu, Femi Igbari, Qiaoliang Bao, Liang-Sheng Liao

**Affiliations:** Jiangsu Key Laboratory for Carbon-Based Functional Materials and Devices, Institute of Functional Nano & Soft Materials (FUNSOM), Soochow University, Suzhou, Jiangsu 215123 China

**Keywords:** Perovskite solar cells, Pump away the solvent, High-quality MAPbI_3 − *x*_Cl_*x*_ layer

## Abstract

A new approach to improve the quality of MAPbI_3 − *x*_Cl_*x*_ perovskite film was demonstrated. It involves annealing the precursor film after pumping away the solvent, which can decrease the influence of solvent evaporation rate for the growth of the MAPbI_3 − *x*_Cl_*x*_ perovskite film. The resulting film showed improved morphology, stronger absorption, fewer crystal defects, and smaller charge transfer resistance. The corresponding device demonstrated enhanced performance when compared with a reference device. The averaged value of power conversion efficiency increased from 10.61 to 12.56 %, and a champion efficiency of 14.0 % was achieved. This work paves a new way to improve the efficiency of perovskite solar cells.

**Electronic supplementary material:**

The online version of this article (doi:10.1186/s11671-016-1467-9) contains supplementary material, which is available to authorized users.

## Background

Organo-metal halide perovskite solar cell, as a rising star in the field of thin-film photovoltaic cells, has drawn much attention, not only due to the superior optical and electrical properties of perovskite materials, such as broad light absorption range [1], low exciton binding energy [2, 3], longer carrier diffusion length [4–6], and higher charge carrier mobilities [7], but also due to its low cost and easy fabrication process. The first work employing perovskite as a light-harvesting material in solar cells was reported by Miyasaka and co-workers in 2009 with an efficiency of only 3.8 % [1]. Almost double of this efficiency (6.5 %) was reported by Park’s research group in 2011 [8]. Due to the dissolubility of perovskite in liquid electrolyte, devices in both works showed poor stability. When a solid-state hole conductor of 2,2′,7,7′-tetrakis(*N*,*N*-di-*p*-methoxyphenylamine)-9,9′-spirobifluorene (Spiro-OMeTAD) was introduced to replace the liquid electrolyte, both stability and performance were improved dramatically [9]. Amazing progress has been made in recent times [5, 10–18] as a champion efficiency over 20 % has been reported for perovskite-based solar cell [19], making it an ideal candidate for next-generation photovoltaic cells. The development of perovskite solar cells came with the challenge of controlling the morphology of the perovskite layer. Such morphology is strongly influenced by several parameters such as fabrication method [5, 12, 13, 15, 20–22], additives [15, 23–26], and annealing process [16, 27–31]. Hence, efforts have been made to improve the morphology of the perovskite layer. For example, Snaith’s group introduced a dual-source thermal evaporation technique [13]. They obtained a much compact and uniform MAPbI_3 − *x*_Cl_*x*_ perovskite layer compared with the traditional one-step spin-coating method. Liang et al. added a small amount of 1,8-diiodooctane (DIO) to the MAPbI_3 − *x*_Cl_*x*_ precursor solution and found that a high-quality MAPbI_3 − *x*_Cl_*x*_ perovskite film was formed with improved coverage and absorption. As a result, the power conversion efficiency (PCE) was increased by 20 % [23]. Yang Yang et al. investigated the influence of annealing conditions on the perovskite layer. They annealed the precursor film in a humid environment (~30 %) which greatly improved the film quality, grain size, carrier mobility, and lifetime. This method produced planar devices with a pretty high PCE approaching 17.1 % [16]. Rira et al. found that a solvent evaporation rate can strongly influence the growth of the MAPbI_3 − *x*_Cl_*x*_ perovskite film. By controlling the solvent evaporation rate, they obtained a MAPbI_3 − *x*_Cl_*x*_ perovskite layer with improved surface coverage. Thus, improved performance was achieved [28].

In this letter, we report a new approach to obtain a high-quality MAPbI_3 − *x*_Cl_*x*_ perovskite layer, by pumping away the solvent of precursor film before annealing to decrease the influence of solvent evaporation rate on the growth of MAPbI_3 − *x*_Cl_*x*_ perovskite film. This approach was proven to be effective as a compact and uniform perovskite film with stronger absorption, fewer crystal defects, and smaller charge transfer resistance. Devices based on this high-quality perovskite film showed enhanced performance compared with the reference device. The averaged efficiency increased from 10.61 to 12.56 % and a champion PCE of 14.0 % was achieved.

## Methods

### Materials

Methylammonium iodide (MAI) was synthesized by reacting 10 ml of hydroiodic acid (57 wt.% in water, Alfa Aesar) with 24 ml of methylamine (33 wt.% in ethanol, Sigma-Aldrich) in ice bath under nitrogen atmosphere with constant stirring. After reacting for 2 h, the resulting white powder of MAI was collected by rotary evaporator at 50 °C. The MAI was dissolved into ethanol and evaporated for further purification. This step was repeated two times, and the MAI powder was finally collected and dried in a vacuum oven at 60 °C for 30 h. Poly(3,4-ethylenedioxythiophene):poly(*p*-styrene sulfonate) (PEDOT:PSS, Clevios AI 4083) and [6,6]-phenyl-C60-butyric acid methylester (PC_60_BM) were bought from Heraeus (Germany) and Nichem Fine Technology Co. Ltd. (Taiwan), respectively. To prepare MAPbI_3 − *x*_Cl_*x*_ (30 wt.%) precursor solution, MAI and PbCl_2_ (Sigma-Aldrich) were dissolved into *N*,*N*-dimethylformamide (DMF) solvent with a molar ratio of 1:1 under constant stirring. The concentration of PC_60_BM solution was 20 mg/ml in chlorobenzene.

### Device Fabrication

**Fig. 1:**
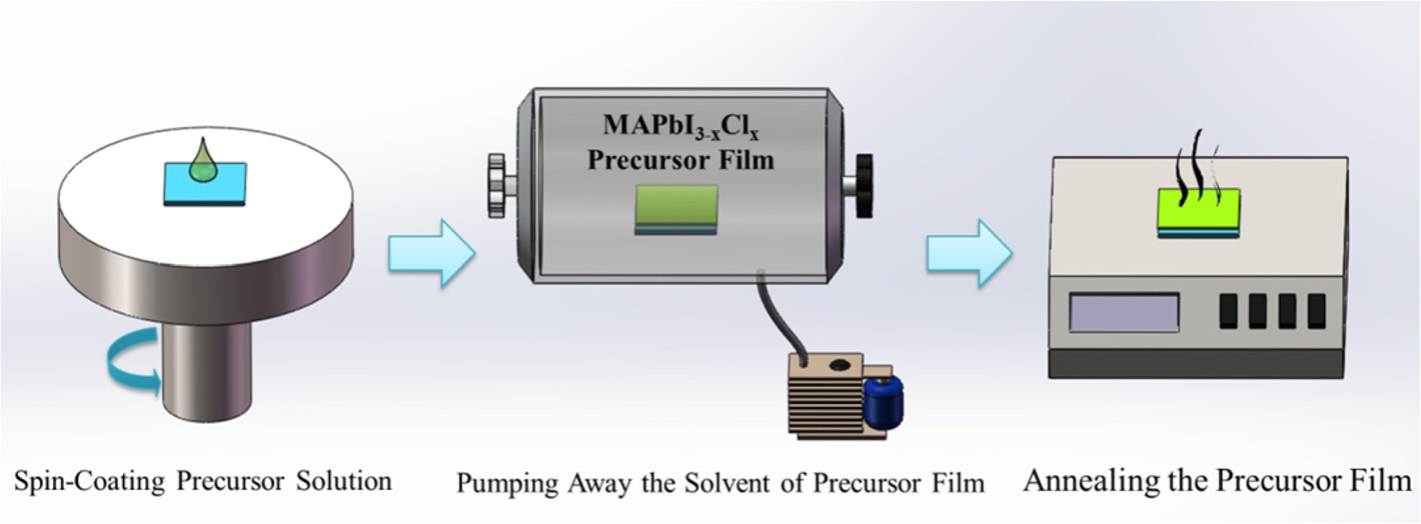
Fabrication process of the MAPbI_3 − *x*_Cl_*x*_ perovskite layer by utilizing our new method

ITO-coated glass substrates with a sheet resistance of ~10 Ω sq^−1^ were sequentially pre-cleaned by acetone, ethanol, and deionized water and dried by oven. After ultraviolet ozone treatment for 15 min, PEDOT:PSS was spin-coated on the ITO surface at 4000 rpm for 40 s. After annealing at 140 °C for 15 min, the substrates were transferred into a nitrogen-filled glove box. The MAPbI_3 − *x*_Cl_*x*_ precursor solution was spin-coated on the PEDOT:PSS surface at 4000 rpm for 40 s. For the reference device, the MAPbI_3 − *x*_Cl_*x*_ perovskite crystal film was achieved by annealing the precursor film directly (100 °C/2 h). While for the modified device, the MAPbI_3 − *x*_Cl_*x*_ perovskite crystal film was achieved by annealing the precursor film after pumping away the solvent, as shown in Fig. 1 (i.e., pumping the solvent component of the precursor film in the transfer chamber of the glove box for 1 h and then annealing at 100 °C for 2 h). As for the solvent removal process, actually, there exists a mechanical pump, which was connected to the transfer chamber. When opening the mechanical pump, the solvent of the precursor film will be pumped away. After cooling down to room temperature, PC_60_BM solution was spin-coated on the perovskite layer at 2000 rpm/30 s without further annealing. Finally, a 100-nm-thick silver cathode was evaporated on top of PC_60_BM by utilizing an OMV-FS300 thermal evaporator. The active area of the devices (7.25 mm^2^) was defined through a shadow mask.

### Measurements and Characterization

Current density-voltage (*J*-*V*) characteristics of perovskite solar cells were measured in air using a programmable Keithley 2400 source meter under AM1.5G solar irradiation at 100 mW/cm^2^ (Newport, Class AAA solar simulator, 94023A-U). The light intensity was calibrated by a certified Oriel Reference Cell (91150 V) and verified with an NREL-calibrated Hamamatsu S1787-04 diode. The external quantum efficiency (EQE) was measured by a certified IPCE instrument (Zolix Instruments, Inc., Solar Cell Scan 100). We utilized a field emission scanning electron microscope (FEI Quanta 200) to investigate the morphology of the perovskite layer. The absorption spectra were measured with a UV/vis spectrophotometer (PerkinElmer Lambda 750). The steady-state photoluminescence spectra were measured by utilizing Horiba Jobin-Yvon LabRAM HR800. Impedance spectroscopy (IS) measurements were performed using a Wayne Kerr 6550B precision impedance analyzer with a 50-mV perturbation oscillation signal in a frequency range from 20 Hz to 20 MHz.

## Results and Discussion

**Fig. 2:**
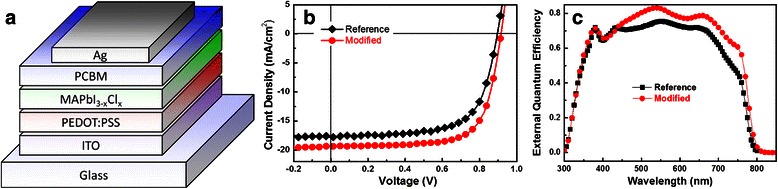
**a** Device structure of the MAPbI_3 − *x*_Cl_*x*_-based perovskite solar cells. **b** Current density-voltage curves of the reference and modified devices measured under AM 1.5G solar illumination at 100 mW/cm^2^. **c** External quantum efficiency of the reference and modified devices

Figure 2a shows the device structure in which PEDOT:PSS and PCBM act as hole and electron transporting layers, respectively. The reference device, in which the MAPbI_3 − *x*_Cl_*x*_ perovskite layer was obtained by directly annealing the precursor film, and the modified device, in which the MAPbI_3 − *x*_Cl_*x*_ perovskite layer was obtained by annealing the precursor film after pumping away the solvent, were both measured in air under AM 1.5G solar illumination at 100 mW/cm^2^. The corresponding current density-voltage (*J*-*V*) curve and photovoltaic parameters are shown in Fig. 2b and Table 1. As presented from the data, the reference device, the MAPbI_3 − *x*_Cl_*x*_ perovskite layer was obtained by directly annealing the precursor film, resulting in an efficiency of 10.61 %. When the MAPbI_3 − *x*_Cl_*x*_ perovskite layer was obtained by annealing the precursor film after pumping away the solvent, an enhanced performance was achieved with a PCE of 12.59 %, corresponding to an open-circuit voltage (*V*_oc_) of 0.91, a short circuit current density (*J*_sc_) of 19.37 mA/cm^2^, and a fill factor (FF) of 0.71. In addition, the modified device showed smaller series resistance (*R*_s_) and larger shunt resistance (*R*_sh_) which is consistent with the increase in FF. The external quantum efficiency (EQE) for both devices was also measured, as shown in Fig. 2c. We can observe that both devices generated photocurrent up to 800 nm, which corresponds to a bandgap of 1.55 eV. The modified device showed an enhanced EQE spectrum in the range of 400–800 nm. The enhanced EQE value is consistent with the increase in *J*_sc_. From the current density-voltage curves of both the reference and modified devices measured in dark condition (Additional file 1: Figure S1), we find that the leakage current of the modified device is almost one magnitude smaller than that of the reference device. This result indicates that more photocurrent was transported to the electrodes in the modified device.

**Table 1:** Photovoltaic parameters of both the reference and modified MAPbI_3 − *x*_Cl_*x*_-based perovskite solar cells

Solar cells	*J*_sc_ (mA/cm^2^)	*V*_oc_ (V)	FF (%)	*R*_s_ (Ω cm^2^)	*R*_sh_ (Ω cm^2^)	PCE (%)
Reference	17.78	0.89	0.67	33.71	2379.16	10.61
Modified	19.37	0.91	0.71	23.51	2921.82	12.59

All the photovoltaic parameters are the average of 12 devices. Series resistance (*R*_s_) and shunt resistance (*R*_sh_) were extrapolated from the slopes of *J*-*V* curves at the open-circuit and short circuit conditions

**Fig. 3:**
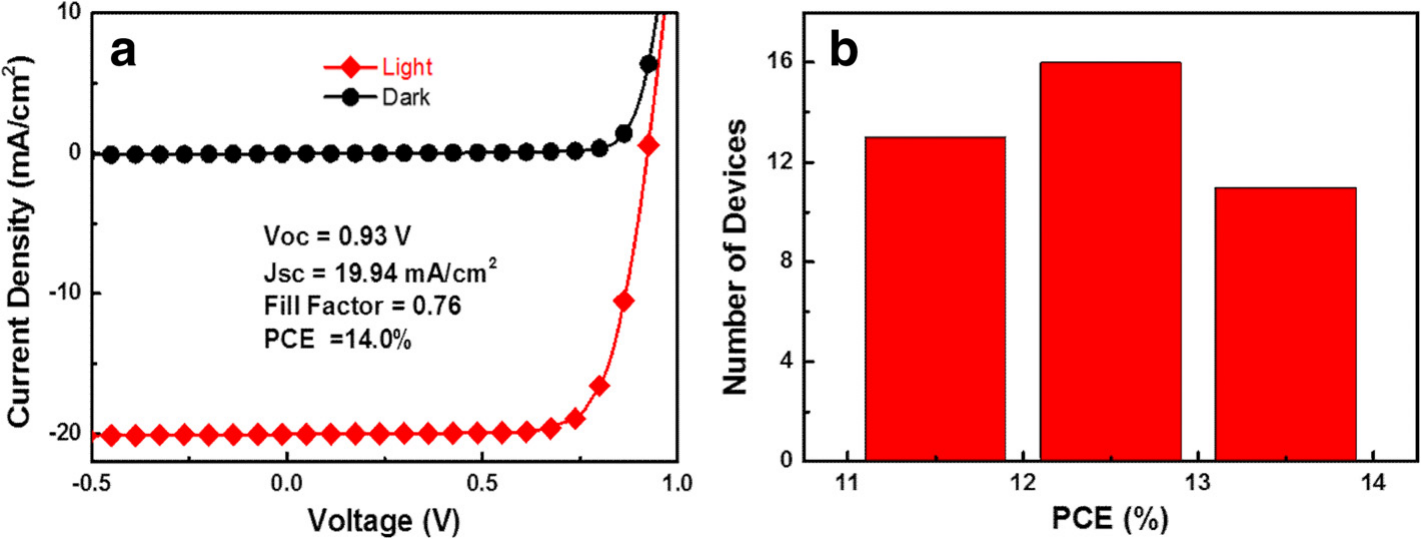
**a** Current density-voltage curves of the champion device. **b** PCE histogram of 40 modified (with pumping) devices

For devices based on the perovskite layer that was annealed after pumping away the solvent of the precursor film, we achieved a champion efficiency of 14.0 % with a *V*_oc_ of 0.93 V, *J*_sc_ of 19.94 mA/cm^2^, and a FF of 0.76 as shown in Fig. 3a. From the PCE histogram of 40 modified devices, shown in Fig. 3b, it is obvious that the modified device also showed good reproducibility. In order to investigate the hysteresis properties of our modified device, we measured a representative modified device in both forward and reverse directions. As depicted from Additional file 1: Figure S2 and Table S1, when the device was measured in the forward direction, a PCE of 12.64 % was achieved. When we changed the scan direction to reverse mode, the device showed an efficiency of 13.58 %. Noticeably, our modified device possesses small hysteresis properties. The hysteresis effect is so small, which is possibly because of the good crystallinity of the MAPbI_3 − *x*_Cl_*x*_ perovskite film. As for the origin of the anomalous hysteresis in perovskite solar cells, three major contributions have been suggested: (a) defects located at surfaces or interfaces of the perovskite materials, (b) ferroelectric properties of perovskite structures, and (c) ion migration due to excess ions that serve as interstitial defects. Even though numerous efforts have been made, it is still not clearly understood presently [32].

**Fig. 4:**
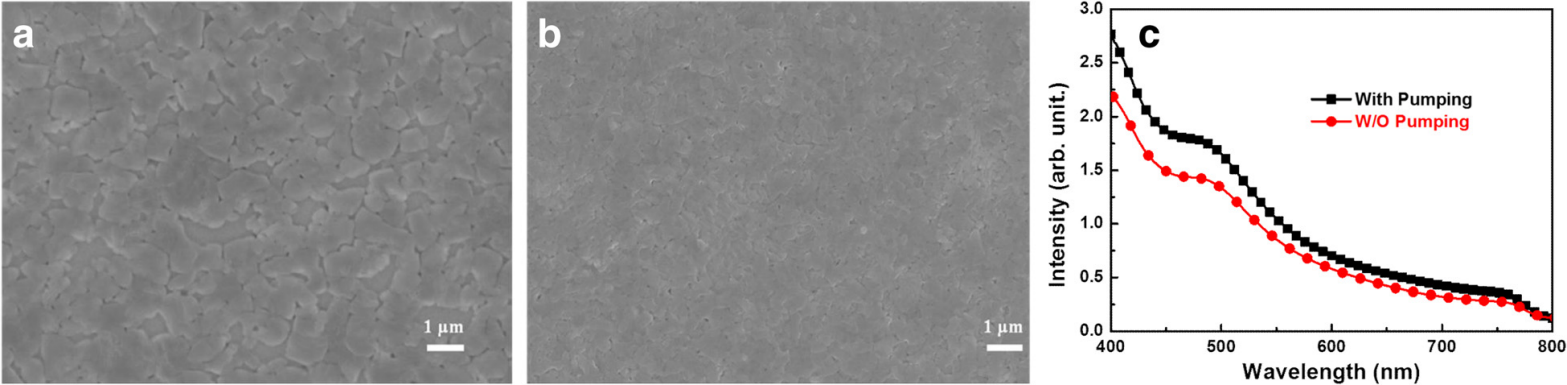
SEM images of MAPbI_3 − *x*_Cl_*x*_ perovskite layers prepared by **a** annealing the precursor film directly and **b** annealing the precursor film after pumping away the solvent. **c** UV-vis absorption spectra of MAPbI_3 − *x*_Cl_*x*_ perovskites prepared by either annealing the precursor film directly or annealing the precursor film after pumping away the solvent

In order to understand the reasons behind the improvement of device performance, we carried out SEM, UV-vis absorption, steady-state photoluminescence spectra, and impedance spectroscopy measurements on the perovskite layers and the corresponding devices. Initially, we investigated the surface properties of the perovskite layers prepared by both methods. The SEM images are shown in Fig. 4. When the MAPbI_3 − *x*_Cl_*x*_ precursor film was annealed directly (Fig. 4a), larger pinholes appeared. We can see that the coverage ratio is pretty low. This will obviously result in poor device performance. When we annealed the MAPbI_3 − *x*_Cl_*x*_ perovskite precursor film after pumping away the solvent, the perovskite layer becomes much more compact and uniform (Fig. 4b). Full surface coverage was obtained, which is beneficial for device performance. We also measured their absorption properties (Fig. 4c). Due to the low coverage ratio of the perovskite layer achieved by direct annealing, light was able to pass through the large pinholes and weaken the absorption. While for the perovskite layer prepared by annealing the precursor film after pumping away the solvent, a stronger absorption was obtained. This distinctly results in higher external quantum efficiency and *J*_sc_, hence an improved device performance.

**Fig. 5:**
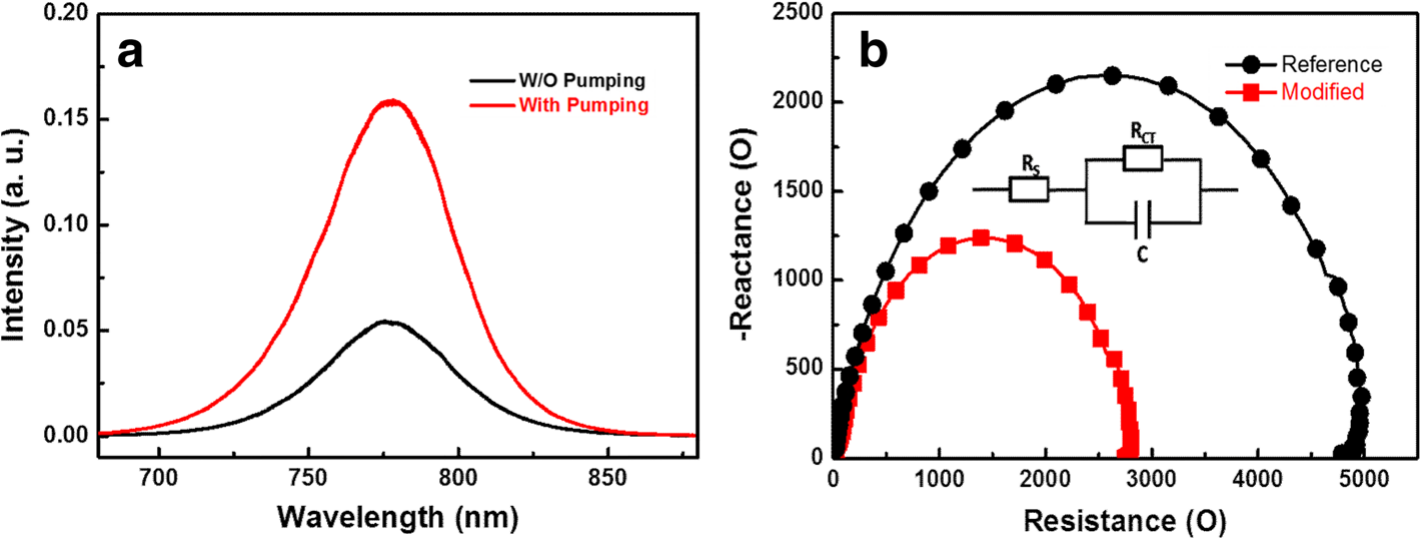
**a** The steady-state photoluminescence spectra of both perovskites on the ITO/PEDOT:PSS surface, prepared by annealing the precursor film directly or by annealing the precursor film after pumping away the solvent. **b** Nyquist plots at *V* ≈ *V*_oc_ for the reference and modified devices with the structure of ITO/PEDOT:PSS/MAPbI_3 − *x*_Cl_*x*_/PC_60_BM/Ag

Figure 5a shows the steady-state photoluminescence spectra of both perovskites on the ITO/PEDOT:PSS surface, prepared by annealing the precursor film directly and by annealing the precursor film after pumping away the solvent. Noticeably, the MAPbI_3 − *x*_Cl_*x*_ perovskite achieved by annealing the precursor film after pumping away the solvent showed much stronger photoluminescence intensity. It implies that the non-radiative decay is significantly suppressed through our new method. This can effectively reduce the number of crystal defects, thus resulting in fewer carrier recombination and better charge extraction.

We also utilized IS to investigate the series resistance (*R*_s_) of both devices. The *R*_s_ consists of sheet resistance (*R*_sheet_) of the electrodes and charge transfer resistance (*R*_CT_). The following three parts contribute to the *R*_CT_: the interfaces between electrodes and charge extraction layer, the interfaces between charge extraction layer, and perovskite layer as well as the bulk of the perovskite layer [33]. Figure 5b shows the Nyquist plots of both devices tested under applied voltage conditions approaching the *V*_oc_ of perovskite solar cells. *R*_s_ values of 4.9 and 2.8 kΩ were obtained for the reference and modified devices, respectively. Noticeably, the modified device showed much smaller *R*_s_. Since the main difference is located at the perovskite layer in this study, it indicates that the MAPbI_3 − *x*_Cl_*x*_ perovskite layer prepared by annealing the precursor film after pumping away the solvent exhibits much smaller *R*_CT_. Enhanced performance was therefore obtained for the modified device.

## Conclusions

A new approach which involves the annealing of a precursor film after pumping away its solvent component was introduced to obtain a high-quality MAPbI_3 − *x*_Cl_*x*_ perovskite film. The device based on such high-quality film showed enhanced performance compared with the reference device. The averaged efficiency increased from 10.61 to 12.56 %, and a champion efficiency of 14.0 % was achieved. SEM, UV-vis absorption, steady-state photoluminescence spectra, and impedance spectroscopy results indicated that the improvement in device efficiency is mainly attributed to the improved morphology, stronger absorption, and fewer crystal defects as well as smaller charge transfer resistance of the modified MAPbI_3 − *x*_Cl_*x*_ perovskite film. This work paves a new way to improve the efficiency of perovskite-based solar cells.

## Additional File

Additional file 1:
*J-V* curves of both the reference and modified devices measured in dark condition. *J-V* curves and photovoltaic parameters of a modified MAPbI_3 − *x*_Cl_*x*_-based device scanned in both forward and reverse directions. Device stability of both reference and modified devices measured in air (humidity: ~40 %, temperature: ~20 °C).
