# Supplementary material for: Efficiency Enhancement of Perovskite Solar Cells by Pumping Away the Solvent of Precursor Film Before Annealing
Source: Nanoscale Res Lett. 2016 May 12;11:248. doi: 10.1186/s11671-016-1467-9 (PMC4864791; doi:10.1186/s11671-016-1467-9)
Supplement: Additional file 1: — J-V curves of both the reference and modified devices measured in dark condition. J-V curves and photovoltaic parameters of a modified MAPbI3 − xClx-based device scanned in both forward and reverse directions. Device stability of both reference and modified devices measured in air (humidity: ~40 %, temperature: ~20 °C). [file 11671_2016_1467_MOESM1_ESM.doc]

**Supporting Information**

Efficiency Enhancement of Perovskite Solar Cells by

Pumping Away the Solvent of Precursor Film before Annealing

Qing-Yang Xu, Da-Xing Yuan, Hao-Ran Mu, Femi Igbari, Qiao-Liang Bao* and Liang-Sheng Liao*

*Jiangsu Key Laboratory for Carbon-Based Functional Materials & Devices, Institute of Functional Nano & Soft Materials (FUNSOM), Soochow University, Suzhou, Jiangsu 215123, China.*

*Corresponding author. E-mail: [qlbao@suda.edu.cn](mailto:qlbao@suda.edu.cn) [lsliao@suda.edu.cn](mailto:lsliao@suda.edu.cn)


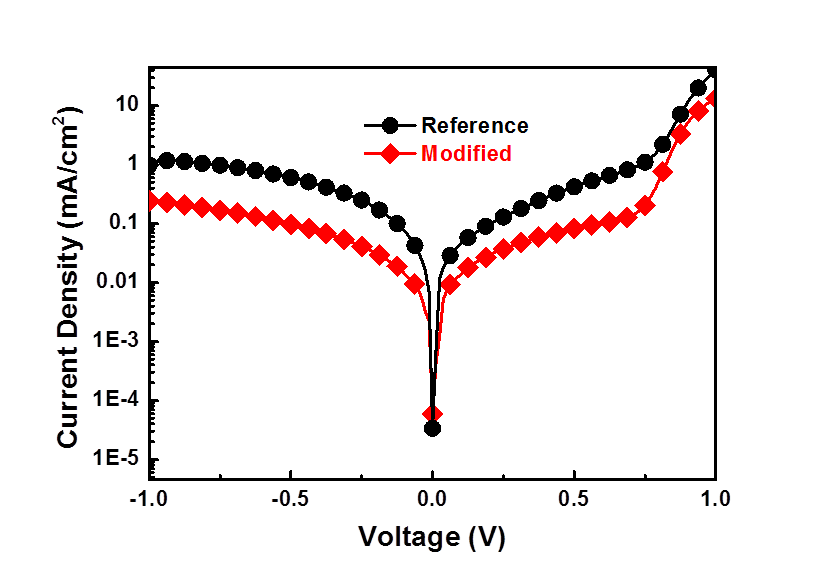


**Figure S1** Current density-voltage curves of both reference and modified devices measured in dark condition. In the reference device, MAPbI_3-x_Cl_x_ perovskite layer was prepared by annealing the precursor film directly. In the modified device, MAPbI_3-x_Cl_x_ perovskite layer was prepared by annealing the precursor film after pumping away the solvent.


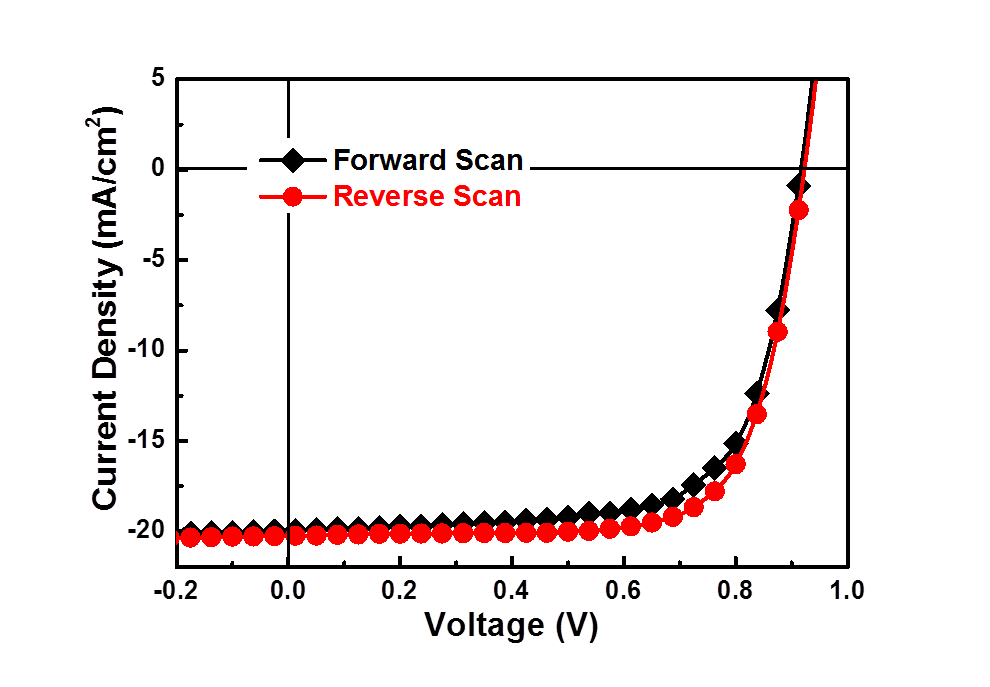


**Figure S2** Current density-voltage curves of a modified MAPbI_3-x_Cl_x_-based device scanned in both forward and reverse directions. The related photovoltaic parameters are summarized in Table S1.

**Table S1** Photovoltaic parameters of the representative MAPbI_3-x_Cl_x_-based modified device scanned in both forward and reverse directions.

| **Solar**  **Cells** | ***J_sc_***  **(mA/cm^2^)** | ***V_oc_***  **(V)** | **Fill Factor**  **(%)** | **PCE**  **(%)** |
| --- | --- | --- | --- | --- |
| Forward Scan | 19.96 | 0.91 | 0.69 | 12.64 |
| Reverse Scan | 20.41 | 0.93 | 0.72 | 13.58 |


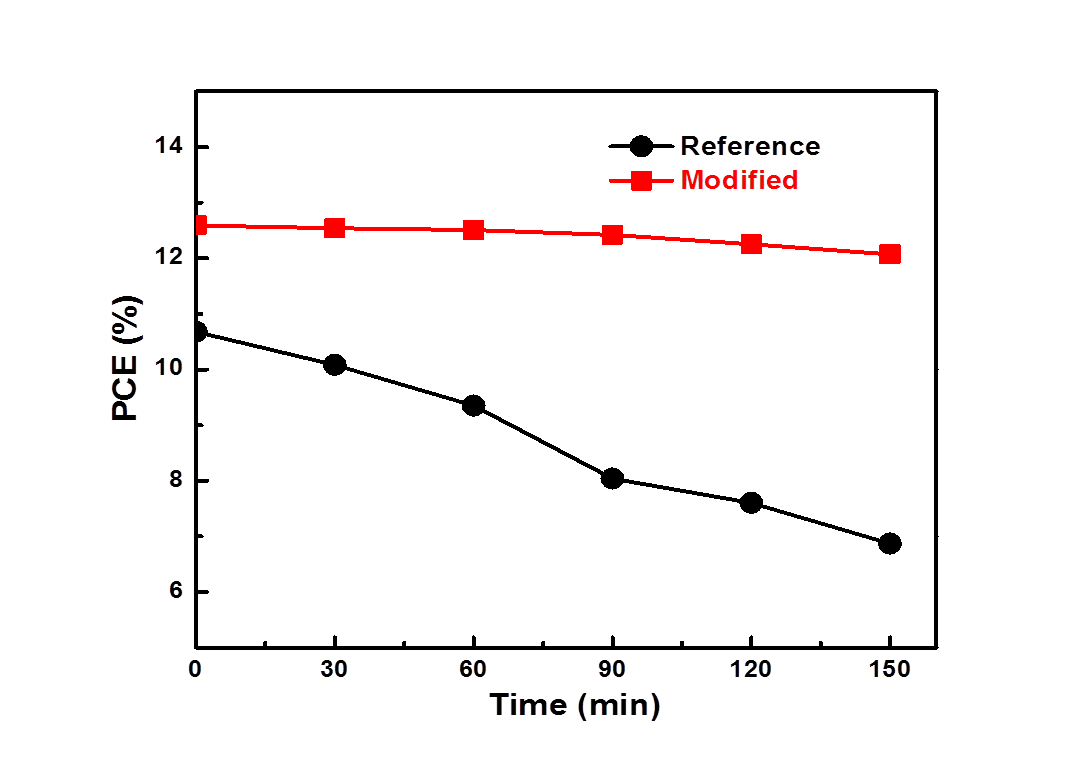


**Figure S3** Device stability of both reference and modified devices measured in air (humidity：~40%, temperature：~20℃).
